# Review of the Nonsteroidal Anti-Inflammatory Drug Consumption, Occurrence, Potential Impacts on Environmental Health, and Insights into Regulatory Decision-Making Brazilian Aquatic Ecosystems

**DOI:** 10.1021/acsomega.5c01916

**Published:** 2025-06-13

**Authors:** Filipe G.A. Godoi, Mariana A. Dias, Cassiana C. Montagner, Fabiana L. Lo Nostro, Renata G. Moreira

**Affiliations:** † Departamento de Fisiologia, Instituto de Biociências, 28133 Universidade de São Paulo, Rua do Matão, Trav.14, n° 321, São Paulo, SP 05508-090 , Brazil; ‡ Laboratório de Química Ambiental, Departamento de Química Analítica, Instituto de Química - UNICAMP, Rua Monteiro Lobado 270, Campinas , SP 13083-862 , Brazil; § Lab. de Ecotoxicología Acuática, Departamento de Biodiversidad y Biología Experimental, Facultad de Ciencias Exactas y Naturales, Universidad de Buenos Aires & IBBEA, UBA-CONICET, Ciudad Universitaria C1428EHA, Buenos Aires 1429 , Argentina

## Abstract

The ubiquitous presence of nonsteroidal anti-inflammatory drugs (NSAIDs) in aquatic compartments has been described, and recent studies reported several adverse biological effects on nontarget species after short- and long-term exposures. Despite the recent reports, integrated information related to the measurements and effects of NSAIDs on Brazilian water ecosystems is still limited, given the importance of Brazilian aquatic biodiversity. Thus, to fill these gaps, after a close literature search using scientific databases, this review aims to summarize the main scientific efforts concerning the occurrence of NSAIDs in Brazilian aquatic environments, the multiple physiological effects on native species, and the different protocols used in the research laboratories. Accordingly to the current literature data (2013–2023), a total of 32 studies were found describing the occurrence of diclofenac, ibuprofen, naproxen, and ketoprofen in Brazilian waters, with concentration ranging from 2.5 to 785,280 ng L^–1^, with the majority of the studies performed in Sao Paulo state (*n* = 10) showing the heterogeneity of monitoring across Brazilian territory. Regarding the adverse effects on native aquatic species, a total of 3 species, including *Rhamdia quelen*, *Astyanax lacustris*, and *Hoplias malabaricus*, have been used to investigate the NSAIDs' adverse effects. The investigations reported endocrine disruption effects by diclofenac and ibuprofen, isolated and combined, in teleosts, oxidative stress responses, and immunotoxicity effects after NSAIDs exposure. When considering the ecotoxicological risk assessment of NSAIDs to Brazilian water bodies, the data showed a low risk quotient (RQ) for the native models across Brazilian territory. However, due to the lack of investigations using representative biological models and robust data concerning the adverse biological impacts of NSAIDs, the RQ may be underestimated, and future directions on NSAIDs investigations are suggested using an integrative approach between environmental safety standards and human health at different environmental risk evaluations.

## Graphical abstract

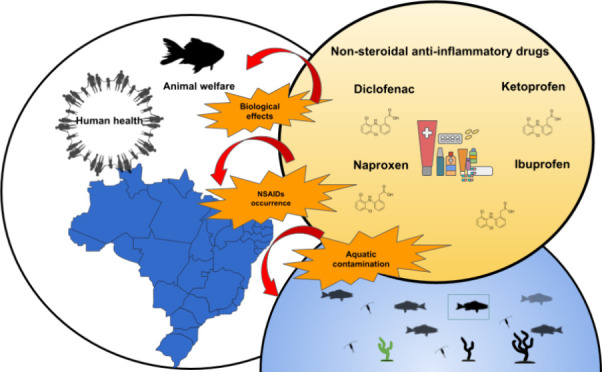

## 1 Introduction

Pharmaceutical active compounds (PhACs) consumption has drastically increased in most populated countries worldwide.^1,2^ Several of these compounds are commonly used in medical treatments for human pathologies and animal diseases, improving the human population’s life expectancy and animal welfare.^3–5^ Moreover, such PhACs acquisition resulted in a growing pharmaceutical market, with an estimated global capital in 2022 of around 1.2 trillion USD.^6^ Despite the economic profits and health benefits, pharmaceutical consumption across the globe culminated in the continuous detection of PhACs in surface, underground, and drinking water, resulting in a growing concern in society.^7–9^

In Latin American (LA) countries, PhACs consumption contributes significantly to the pharmaceutical industry, especially in Brazil. This upper-middle income developing country is one of the major contributors to pharmaceutical consumption in LA^10^ and accounts for a market capital around 25 billion USD.^11^ Nonetheless, as occurs in other geographical regions, PhACs quantification in LA waters has been described in recent literature^12^ and also restricted to specific areas, mainly countries like Brazil and Mexico, followed by Argentina, Colombia, and Chile.^13^ Besides, compared to developed nations, the monitoring programs regarding contaminants of emerging concern (CECs) like the PhACs are still lacking in most LA regions.^13,14^ Distinct causes, including the absence of specific legislation, limited investments in analytical equipment,^14,15^ and a minority of risk assessment procedures, could be linked with the limited available data in LA.^13^

The CECs occurrence in most of the LA countries describes an alarming amount of distinct PhACs presence in natural waters, including β-blockers, hormones, analgesics, lipid regulators, antidepressants, antibiotics, and nonsteroidal anti-inflammatory drugs (NSAIDs).^13,15^ For example, the detection of NSAIDs in LA sampling investigations accounted for approximately 70% of the detection frequency.^13^ Additionally, specific causes such as economic growth and population aging associated with the flexible acquisition of pharmaceuticals, incorrect disposal, also contribute to this new consumption behavior, resulting in more environmental impacts.^13,15^ Considering the presence of NSAIDs in LA waters and understanding the importance of Brazilian aquatic ecosystems, this review aims to describe the pharmacological features and general information regarding the structure, toxicity, function, and pharmacological activity of NSAIDs. Moreover, this review focuses on understanding the consumption trends of NSAIDs in Brazilian territory, summarizing the main scientific efforts concerning their occurrence in Brazilian aquatic environments, identifying the multiple adverse effects on native species, and comprehending the distinct scientific approach in laboratory assessment studies.

First, regarding the absence of integrative data associated with the NSAID occurrence in distinct water matrices, an advanced search was performed to identify the publications containing the occurrence of NSAIDs in Brazilian waters in ten years (2013–2023). For this purpose, advanced search tools, Web of Science, PubMed, and Scopus databases were used, and keywords were chosen as follows: “occurrence” AND “NSAIDs” AND “Brazil” AND “water” plus the name of each chemical. Additional filters included the English language and types of documents, such as original articles and reviews. Secondly, considering the occurrence of NSAIDs in Brazilian water matrices and the continued data of NSAIDs effects on aquatic species across the planet,^16,17^ the present review intends to provide the information about the adverse biological effects on native Brazilian fish species caused by NSAIDs, including endocrine disruption, oxidative stress, developmental abnormalities, survival, and the effects of mixtures, as well as the significant gaps and future directions in the laboratory research and major scientific gaps regarding physiological end points.

## 2 Overview of NSAID Consumption in Brazilian Territory, Chemical Properties, and Pharmacological Mode of Action

### 2.1 NSAID Consumption in Brazilian Territory

Given the importance of the Brazilian pharmaceutical industry for the LA region and the globe, recent investigations focus on understanding the consumption trends and how medicines are acquired by consumers.^18,19^ In Brazil, many PhACs, such as synthetic hormones, NSAIDs, and stimulants, can be sold at drugstores without any physician prescription,^20^ which results in indiscriminate usage and self-medication by the majority of the population.^20,21^ NSAID consumption is also associated with the fact that such chemicals are more accessible^19^ and also offer a fast physiological response after taking NSAID medicines to reduce the pain and the distinct symptoms of diseases.^22^

Regarding NSAID consumption trends, the overall information concerning the acquisition and consumption by the Brazilian population is not informed by the government’s official reports. Still, most of the data instead is related to the revenue of the pharmaceutical industry report, which is provided by the *Secretaria Executiva da Câmara de Regulação do Mercado de Medicamentos* (SCMED), a government division responsible for the pharmaceutical market statistics in Brazilian territory and managed by *Agência Nacional de Vigilância Sanitária* (ANVISA). According to the SCMED annual report,^11^ the medicines industry income increased by 24.1% comparing 2022 to 2020, and also pharmaceuticals without any prescriptions accounted for a 43.1% increase comparing the year 2022 to 2020, in which NSAIDs are included. Moreover, the same report also highlighted that analgesics are first in the total sales, amounting to around 18.5%. These numbers suggest an expansion in the pharmaceutical market, including medicines such as NSAIDs obtained without any medical prescription in Brazilian territory.

Concerning the limited information on Brazilian official numbers, Brazilian databases describing the daily consumption of PhACs without prescription are scarce, forcing different studies to obtain information using nongovernmental databases of survey companies such as IQVIA^23^ and other efforts, such as surveys to gather more information from the population’s medicine consumption behavior.^18,19^ Based on the nongovernmental information and recent literature investigations, the acquisition and consumption of NSAIDs across the Brazilian territory is not uniform. For example, one of the highest demographic densities in Brazil, the São Paulo Metropolitan area, with approximately 22 million people, displays a consumption of 300 types of medicines of 26 therapeutic classes, in which NSAIDs account for 44.3% of the total.^24^ On the other hand, data regarding the NSAID acquisition is limited in different regions of the Brazilian territory, or even nonexistent. The restricted data found in the literature also brings questions associated with the overconsumption and self-medication by the Brazilian population, and recent studies attempt to evaluate and promote the usage of NSAIDs and analgesics among the consumer population.^18,19^

A national survey including the five Brazilian demographic regions reported that most Brazilian consumers of analgesics are women (52.8%) between the ages of 20 and 59 years (57.2%).^19^ In this survey, diclofenac accounts for 10.7% of NSAIDs and analgesics ingested; the study also concluded that one out of five Brazilians uses some analgesic for pain relief and acute health problems. However, the same investigation only displayed the national data and was not separated by geographic region.^19^ Additional surveys conducted by Quadra and colleagues^19^ also described a considerable amount of pain-relief substances consumed by the population, of which 13% were NSAIDs and 30% were analgesic compounds. The study also generated questionnaires with several questions containing the following topics: age, scholarly, living place, most consumed PhACs, disposal medicines, knowledge of medicine disposal, etc. The research results revealed that 66% of the participants discarded PhACs in the garbage; also, the authors suggest that these results elucidate the missing information on PhACs use and disposal by the Brazilian population. Even though most of the interviewed Brazilians (95.2% of the participants) consider that PhAC residues can be harmful to natural environments, around 71.9% of them were not instructed on how medicine compounds should be correctly disposed of.^19^

Contrary to the limited information associated with the consumption by official governmental sources in Brazilian territory,^23^ the national consumption trends of NSAIDs in other geographical regions, including the USA, Asia region, and the European Union, display additional data and it may support future policies in Brazil offering distinctive strategies to acquire monitoring tools to the purchase and use of NSAIDs and other pharmaceuticals. In the USA, studies using the National Health and Nutrition Examination Survey (NHANES) provided by the CDC-USA government observed an increase over time in NSAIDs consumption, mainly in the elderly population, women, and those with higher body mass.^25^ In Asian countries like China, investigations also reported an increase in NSAIDs consumption, including the dosages in different hospitals.^26^ In the European Union, a study conducted in the French population observed an NSAIDs consumption increase of 140% since 2008.^27^ Thus, the NSAIDs data across the distinct geographical regions displayed an increase in usage throughout the years, and also, distinct approaches have been made by the authorities to analyze the side effects of NSAID consumption in different situations, including hospitals and domestic use.

### 2.2 NSAID Pharmacological Mode of Action

NSAID drugs which includes diclofenac, ibuprofen, naproxen, and ketoprofen are frequently used to treat many diseases around the globe, including arthrosis, rheumatoid arthritis, osteoarthritis and used as chronic pain relief and analgesic^28–30^ and even investigations suggest their regulation on COX activity in cancer therapy,^31^ which shows their relevance to human health.^28^ Pharmacologically, NSAIDs are used as inhibitors of prostaglandin Endoperoxide H Synthases (PGHS), commonly known as cyclooxygenase enzymes (COX). Some of these enzymes are responsible for the subsequent enzymatic cascade that begins with the conversion of Arachidonic acid (AA) into prostaglandins (PGs) molecules, which in turn are important in several biological functions such as inflammation, metabolism, and reproduction.^32,33^ The COX enzymes display two specific sites as cyclooxygenase and peroxidase. The oxygenase site, where AA is converted to prostaglandin G2 (PGG_2_), and the peroxidase activity of prostaglandin G/H synthase 2 (PTGS2) catalyzes the reduction of PGG2 to PGH2 using two electrons.^34^ Most mammals and vertebrates express the COX1 isoform, but COX2 is most expressed in specific physiological situations, such as inflammation and stressful metabolic conditions.^35^ The enzymes are also rate-limiting in the conversion of AA to prostanoids.

### 2.3 NSAID Chemical Properties

With regard to NSAID classification, these chemicals are divided according to their structure, solubility (Table 1), half-life, pharmacological activity, and selectivity in the COX inhibition process. NSAIDs found on the market are classified as salicylic acid derivatives (e.g., acetylsalicylic acid), anthranilic acid derivatives (e.g., diclofenac), aryl and heteroaryl acetic acid derivatives (e.g., ibuprofen and naproxen), enolic acid derivatives (e.g., meloxicam), and indole and indene acetic acid derivatives (e.g., indomethacin).^28^ These compounds also display different pharmacological activity on the COX subtype enzymes, where the highest selectivity for COX-2 was observed in etoricoxib, rofecoxib, and valdecoxib compounds. Meanwhile, piroxicam showed the highest affinity to the COX-1 isoform, followed by ibuprofen and naproxen.^32^ The chemical nature of NSAIDs and pharmacological activity is a complex topic of discussion, and there are already specific chemical reviews in the literature.^28,32^

**Table 1:**
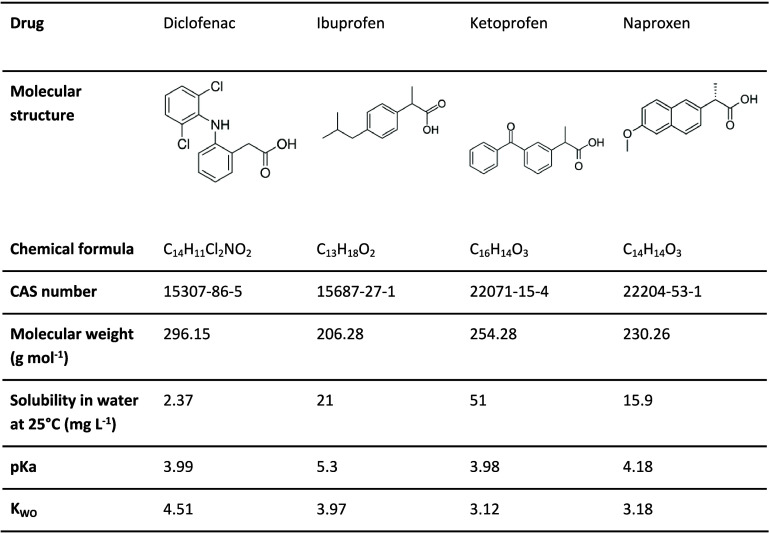
Physical-Chemical Properties of the Main Nonsteroidal Anti-Inflammatory Drugs (NSAIDs) Used in Brazil (Source: PubChem)

An additional challenge associated with NSAID consumption is the byproducts of metabolization,^17^ as metabolized compounds may display higher toxicity compared to the parental molecules.^3^ After ingestion, NSAIDs are absorbed by the intestinal mucosa and redistributed in the body, where aromatase enzymes (CYP) will transform the NSAIDs into different metabolized compounds as hydroxyl components, including 4-hydroxy diclofenac (4-OHDCF) and acyl groups (glucuronides) of diclofenac and ibuprofen.^28^ The metabolization process of each NSAID depends on the molecular structure,^36^ and similar metabolization processes are also observed in wild animals.^37^ In mammals, NSAIDs have been associated not only with gastrointestinal toxicity for humans and domestic animals but also cause reproductive and metabolic disorders.^36,38^ In laboratory conditions, NSAIDs’ long-term exposure in rats resulted in high toxicity not only for the kidneys and liver but also for the reproductive system.^36^

## 3 Occurrence of NSAIDs in Brazilian Waters

In Brazil, as highlighted in recent literature, general reviews related the common causes of PhACs occurrence and persistence in the Brazilian aquatic ecosystems to aquatic pollution.^39–41^ First, the overall consumption and wrong disposal of pharmaceuticals have occurred in the Brazilian territory^,^.^10,18^ Second, there is an increase in the demand for developing and applying specific technologies in wastewater treatment plants (WWTP) for PhACs degradation.^12,23^ Finally, a lack of Brazilian regulatory policies and absence of environmental legislations containing the limits of PhACs on surface waters have been highlighted, which in turn results in poorly monitored programs for water environmental safety.^10,12^ Furthermore, according to the Brazilian Ministry of Regional Development, almost 50% of the Brazilian population does not have access to waste treatments,^42^ leading to even greater pressure on WWTPs to efficiently remove PhACs from domestic waste, hospital effluents, and industry discharges.

Even with an NSAID detection frequency of 70% in Latin America,^13^ in most developing countries, specific legislation for PhACs monitoring programs is still restricted.^12^ Meanwhile, the European Union (EU) already has monitoring programs that evaluate CECs’ impacts on the aquatic environment, followed by the subsequent environmental risk assessment protocols to implement safe limits to PhACs occurrence.^43,44^ For example, the NSAID diclofenac was added to the watch list of the EU directive framework in 2015, but due to the increasing information regarding diclofenac toxicity, in 2018, the compounds were taken out.^45,46^ However, NSAIDs are still under investigation with nonstandardized species due to the effects and missing information related to ibuprofen, naproxen, and ketoprofen.^17^ In the USA, diclofenac is one of the pharmaceuticals classified as CECs and a matter of concern.^47^

From a global perspective, the occurrence of NSAIDs is diverse when considering the dispersion, detection, and environmental limits established across different geographical regions described in the literature reviews.^48^ The concentration changes across continents, beginning in 0.6 ng L^–1^ in North America to 17,400 ng L^–1^ in Africa,^7,9,16^ also Europe to Asia, ranging from 4.62 ng L^–1^ to over 836 μg L^–1^.^49^ Additionally, investigations into the occurrence of NSAIDs in the surface and underground waters have been conducted mainly in developed countries, resulting in unbalanced data related to NSAID environmental toxicology in the literature. Moreover, the information regarding NSAIDs’ quantification and distinct effects on biota is still limited.^16^

In Brazil, the occurrence of NSAIDs is observed from the nanogram to microgram concentration scale, but the detection values are not equally reported due to the lack of data in many areas of intense human activity (Figure 1). The most populated Brazilian states, São Paulo, Minas Gerais, and Rio de Janeiro, account for most of the present data related to NSAIDs occurrence in Brazilian freshwater and seawater. Thus, the Southeastern region displayed more information related to the concentrations of NSAIDs in water (Figure 1 and Table 2). This missing information is mainly linked to many factors, such as monitoring programs, research facilities, and financial resources. In São Paulo state, for example, most research comes from universities that provide more infrastructure to measure these compounds.^50^

**Table 2:** Occurrence of Main Nonsteroidal Anti-Inflammatory Drugs (NSAIDs) on Water Matrices from Different Sampling Sites on the Brazilian Territory

Sampling site		Chemical	Concentration range (ng L^–1^)	Frequency of detection and number of samples	Ref.
Southeastern region	surface water from Piraí and Jundiaí rivers (Jundiaí River Basin-SP)	diclofenac	9.11–328.5	79% (*n* = 42)	^58^
		ibuprofen	3.33–208.2	69% (*n* = 42)	
		naproxen	5.14–98.6	93% (*n* = 28)	
	surface water from Piraí and Jundiaí rivers (Jundiaí River Basin-SP)	diclofenac	4.88–364	100% (*n* = 24)	^59^
		ibuprofen	6.75–373	100% (*n* = 24)	
		naproxen	5.67–145	100% (*n* = 24)	
	surface water from urban drainage channel in São Vicent Island (SP)	diclofenac	1.1–2.5	100% (*n* = 5)	^60^
	surface water from urban drainage channel in Santos beaches (Santos-SP)	diclofenac	1.9–3.5	100% (*n* = 7)	^61^
	surface water from urban drainage channel in Guarujá beach (SP)	diclofenac	0.9–79.8	100% (*n* = 8)	^62^
	surface- and bottom water around the coastal submarine sewage outfall in Guarujá (Santos-SP)	diclofenac	3.6–85.7	75% (*n* = 8)	^63^
	surface water from coastal rivers in São Paulo coast (SP)	diclofenac	0.76–3.93	100% (*n* = 5)	^64^
	surface- and bottom water from Santos Bay (Santos-SP)	diclofenac	19.4^a^	100% (*n* = 10)	^65^
		ibuprofen	326.1–2094.4	100% (*n* = 10)	
	surface water from Lobo reservoir (Itirapina-SP)	diclofenac	50^b^	71.4% (*n* = 30)	^66^
		ibuprofen	130^b^	42.8% (*n* = 30)	
		naproxen	100^b^	85.7% (*n* = 30)	
	surface water from Monjolinho River (São Carlos-SP)	diclofenac	22.4–385.6	60% (*n* = 126)	^67^
		ibuprofen	45.7–743.9	60% (*n* = 126)	
		naproxen	2.9–655.2	60% (*n* = 126)	
	surface water from Itaipu-Piratininga coastal lagoons (Niterói-RJ)	ibuprofen	27.6–37.6	50% (*n* = 4)	^68^
		naproxen	16.3–22.5	50% (*n* = 4)	
	surface water from rivers (Rio de Janeiro-RJ)	diclofenac	220^a^	20% (*n* = 5)	^69^
	surface water from João Mendes River basin (Niterói-RJ)	ibuprofen	1300–10,700	100% (*n* = 16)	^70^
	surface water from Paraopeba River Basin (MG)	diclofenac	136.6–2625.7	100% (*n* = 12)	^71^
	surface water from Paraopeba River Basin (MG)	diclofenac	6.3–561.0	30% (*n* = 60)	^72^
		ibuprofen	5.6–1683.9	62% (*n* = 60)	
		naproxen	4.7–938.4	87% (*n* = 60)	
	water from drinking water treatment plants (MG)	ibuprofen	302–333	47% (*n* = 152)	^51^
		ketoprofen	22–1020	92% (*n* = 152)	
	surface water from water supply systems (Belo Horizonte-MG)	diclofenac	1115.2^b^	6% (*n* = 252)	^73^
		ibuprofen	1629.2^b^	65% (*n* = 252)	
		naproxen	26,566.2^b^	23% (*n* = 252)	
Southern region	surface water from Alto Iguaçu watershed (Curitiba-PR)	diclofenac	34–285	90% (*n* = 120)	^74^
		ibuprofen	102–370	50% (*n* = 120)	
	surface water from Iguaçu River (Curitiba-PR)	ketoprofen	620^b^	18% (*n* = 64)	^54^
		naproxen	340^b^	34% (*n* = 64)	
	surface water from Tibagi River (PR)	diclofenac	8.0–375.0	46% (*n* = 13)	^56^
		naproxen	62.6–1566.4	54% (*n* = 13)	
	surface water from Lake Guaíba (Porto Alegre-RS)	diclofenac	29–107	83% (*n* = 35)	^55^
		ibuprofen	274–387	14% (*n* = 35)	
		ketoprofen	<21.0	43% (*n* = 35)	
		naproxen	<21.0	17% (*n* = 35)	
	surface water from urban rivers (Porto Alegre-RS)	diclofenac	<1.0	52% (*n* = 54)	^75^
		naproxen	<1.0	22% (*n* = 54)	
	surface water from Cancela-Tamandaí and João Goulart watershed (Santa Maria-RS)	ibuprofen	200–2710	85% (*n* = 20)	^76^
	estuarine water from Santa Catarina coastal area (SC)	diclofenac	1.40–7.92	100% (*n* = 8)	^77^
Midwestern region	surface water from Stream of Onça (Três Lagoas-MS)	diclofenac	120–8250	89% (*n* = 72)	^78^
		naproxen	80–21,285	58% (*n* = 72)	
	surface water from streams in Dourados (MS)	diclofenac	7–849	90% (*n* = 84)	^79^
		naproxen	9–681	97% (*n* = 84)	
	drinking water (Brasília-DF)	diclofenac	4.2–6.03	14% (*n* = 14)	^52^
		ibuprofen	4.0–4.8	29% (*n* = 14)	
Northeast region	surface water from Beberibe River Basin (Recife-PE)	diclofenac	19–193,000	100% (*n* = 12)	^80^
	surface water from São Francisco River (PE)	diclofenac	607,920–759,060	76% (*n* = 36)	^53^
		ibuprofen	88,020–785,280	67% (*n* = 36)	
	surface water from Anil and Bacanga rivers (São Luis-MA)	diclofenac	105–463	36% (*n* = 28)	^81^
		ibuprofen	113–320	54% (*n* = 28)	
Northern region	surface water from Igarapé do 40, Igarapé Mindu and Rio Negro (Manaus-AM)	diclofenac	63–785	44% (*n* = 16)	^82^
	water from Bolonha Water Treatment Plant (Belém-PA)	ibuprofen	9.1^a^	8% (*n* = 12)	^83^
		naproxen	351.8^a^	8% (*n* = 12)	

a Single concentration.

b Maximum concentration.

**Figure 1:**
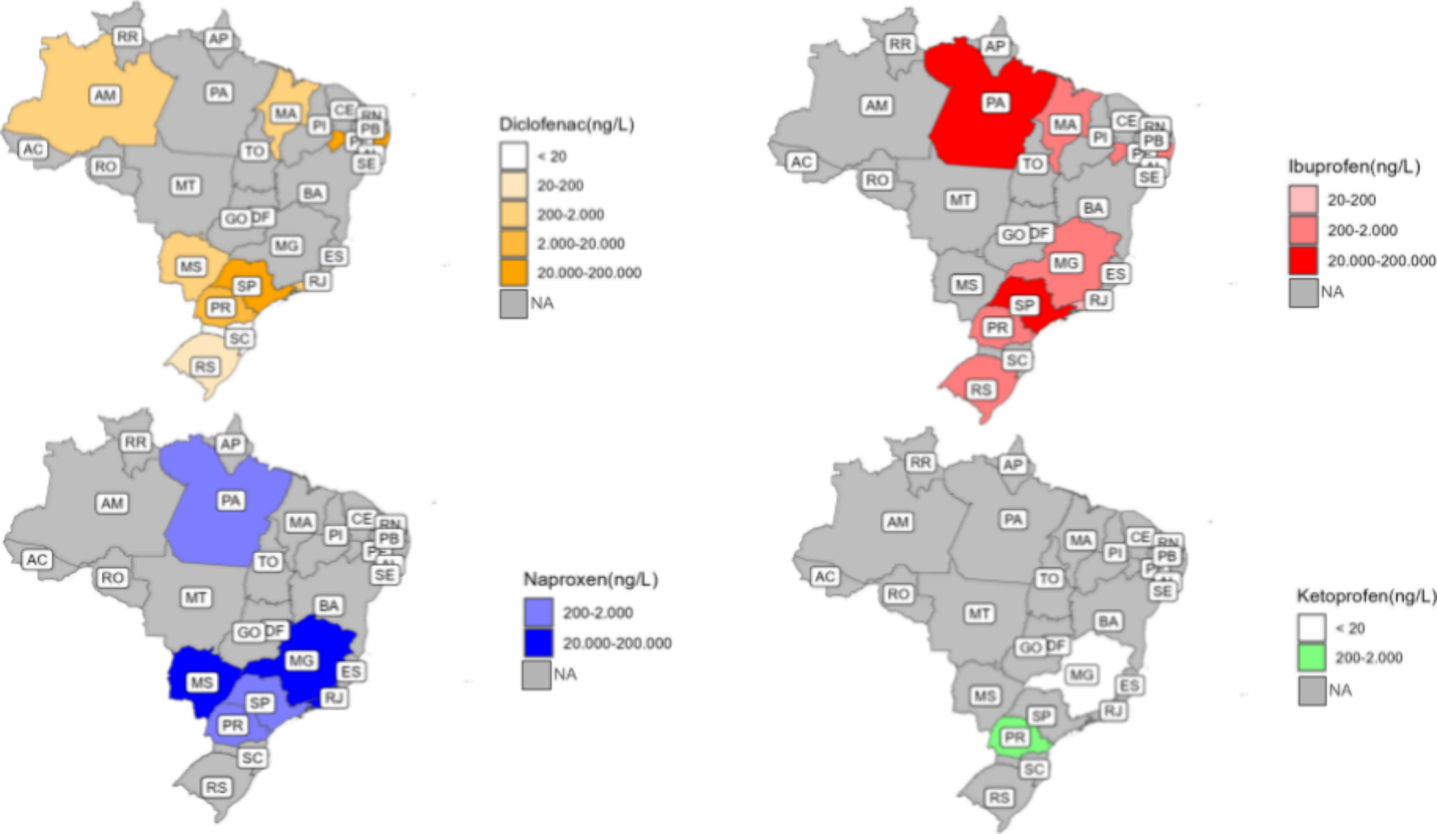
Nonsteroidal anti-inflammatory drug (NSAID) detection range (ng L^–1^) in the Brazilian states aquatic ecosystem.

In the present review, after performing an advanced search to identify the articles containing the occurrence of NSAIDs in Brazilian waters in the last ten years (2013–2023), we found 32 papers describing the concentrations of NSAIDs, as shown in Table 2. According to the information obtained in those articles, 10 of the studies were from São Paulo state, with NSAID maximum concentrations ranging from 2.5 to 2094.4 ng L^–1^. Seventeen publications were found in southeastern Brazil, becoming the region with the most data on NSAID occurrence. Also, most studies were focused on surface waters; a total of 30 of the 32 publications elucidated the occurrence in surface waters, and only 2 of them were targeted for drinking water, where concentration ranged from 4.8 ng L^–1^ of ibuprofen to 1020 ng L^–1^ of ketoprofen, especially from treatment plants.^51,52^

Ibuprofen and diclofenac are the most common NSAIDs studied in Brazil, their concentrations ranging from 3.33 to 785,280 ng L^–1^ and 0.76–759,060 ng L^–1^, respectively. The highest ibuprofen and diclofenac concentrations were found in surface water from the São Francisco River.^53^ Fourteen papers studied the naproxen occurrence in Brazilian aquatic matrices, where its concentration ranged from <1.0 to 26,566.2 ng L^–1^. In addition, only 3 papers identified ketoprofen in water samples with concentrations of 620 ng L^–1^ in surface water from the Iguaçu River,^54^ 22–1020 ng L^–1^ in drinking water from Minas Gerais water treatment plants,^52^ and <21.0 ng L^–1^ in surface water from Lake Guaíba.^55^

Another critical information retrieved from the NSAIDs quantified across Brazilian rivers is associated with the different NSAID concentration values found in the northeast and southern regions. Comparing the maximum diclofenac values of 9375 ng L^–1^ in the Tibagi River^56^ against the concentration of 759,060 ng L^–1^ in the surface waters of the São Francisco River.^53^ These values may also elucidate the lack of basic sewage treatment, resulting in possible impacts on environmental health and a decrease in the quality of water resources for human and animal consumption in those regions. Although NSAIDs display pseudo persistence due to their continue input in the aquatic environment and most of the WWTP across the country are not able to reduce a certain due to the limited technology available in the treatment plants,^12,20^ efforts have been made to reduce the presence of these chemicals using the active sludge and membrane bioreactor^57^ and also understand the consumption trends of PhACs, including NSAIDs, to incentive new educational programs in the use of pharmaceutical products.^18^

In addition to the occurrence of diclofenac, ibuprofen, naproxen, and ketoprofen (Table 1), the distribution of these PhACs along the country’s territory is also shown on the graphical maps (Figure 1). Brazil’s southeastern and southern regions account for most of the research in the environmental toxicology area, mainly due to the resource funds^50^ and affordable studies that can be conducted in these areas. Although other geographic regions showed fewer data compared with southeastern states, there are a few areas in the northeast and northern regions, including Pernambuco, Maranhão, and Pará states, in which the quantification of NSAIDs was measured. Much of diclofenac and ibuprofen was found in higher concentrations, ranging from 19 to 785,280 ng L^–1^, in surface waters of relevant rivers for the populations, including the São Francisco River.^53^

## 4 Biological Effects of NSAIDs on Brazilian Aquatic Species

The NSAIDs application in human health and veterinary medicine has become an affordable way to minimize pain-related diseases worldwide.^28^ Nevertheless, the increasing consumption associated with inappropriate discharge and the inability of WWTPs to retrieve such chemicals^7,9^ resulted in the presence of NSAIDs in the water bodies across the globe, including Brazilian aquatic ecosystems, posing a threat to aquatic organisms and also to humans. According to the World Health Organization (WHO), understanding the adverse effects of anthropogenic stressors using a One Health approach offers reliable tools to protect and mitigate the impacts of human activities.^84^ This approach is associated with a cross-disciplinary interface between animals, environment, humans, and the complex aquatic biodiversity in Brazilian ecosystems.^85^ Distinct studies have focused on understanding how endemic species respond to NSAID exposure. Studies linking the pharmaceuticals as NSAIDs’ adverse effects on Brazilian native species are still limited or even nonexistent, considering most aquatic species that are part of the Brazilian biota.^14,86^

Taking the few earlier reports, most of the observed effects are related to oxidative stress, reproduction, development, and endocrine disruption. To the best of our knowledge, only three native teleost species were used to assess the effects of NSAIDs on the Brazilian ecosystem, *Rhamdia quelen*,^87–89^ *Astyanax lacustris* (previous classified as *Astyanax altiparanae*),^86,90–93^ and *Hoplias malabaricus* ^94,95^ and more recently a field study evaluating bioconcentration and oxidative stress biomarkers including SOD, CAT and GPx responses in endemic oysters *Crassostrea gasar*.^96^

In Brazilian territory, even considering the widespread presence of such contaminants in surface waters, only two research groups in Brazil investigated the capacity of NSAIDs to impact the endocrine activity of native Brazilian aquatic species, as shown in Table 3. From a scientific perspective, the main investigations elucidate that NSAIDs display distinct toxicity levels to standardized laboratory species such as zebrafish *Danio rerio*. The common effects are linked to redox imbalance, developmental abnormalities, endocrine disruption responses,^16^ changes in behavior, and even genetic modifications have also been described using freshwater invertebrates^17^ and modulation of physiological end points in marine bivalves.^97^ Considering the negative ecological impacts observed in terrestrial vertebrates such as vultures,^98^ chronic NSAIDs exposure might also impact the biodiversity of aquatic species,^16,17^ and the limited data in certain geographical regions, such as LA, may not disclose several problems into the real threats caused by such contaminants.^99^ In comparison to the LA region, developed countries’ policies related to environmental safety are more effective in quantifying these xenobiotics in the environment,^100^ but in other developing countries from LA, including Brazil, this information is still limited, even considering the importance of biodiversity protection.^12,99^

**Table 3:** Biological Effects in Fish After Nonsteroidal Anti-Inflammatory (NSAID) Drugs Exposure

Compound	Experimental condition	Concentration	Species	Biological effects	Ref.
diclofenac	laboratory	0.2, 2, and 20 μg L^–1^	*Rhamdia quelen*	increase of superoxide dismutase activity (SOD) in kidney	^87^
diclofenac	laboratory	3.08 mg L^–1^	*A. altiparanae*	reduction of testosterone (T) and 17β-Estradiol (E_2_) concentration	^90^
diclofenac ibuprofen	laboratory	3.08 and 13.7 mg L^–1^	*A. altiparanae*	increased thyroxine (T4) in 24 h	^86^
				decreased triiodothyronine (T3) concentration at 96 h	
diclofenac	laboratory	0.2, 2, and 20 μg L^–1^	*Rhamdia quelen*	reduction of dopamine and metabolite DOPAC	^88^
				effects of antioxidant defense on liver and gonads	
ibuprofen	laboratory	0.1, 1, and 10 μg L^–1^	*Rhamdia quelen*	disturbance on the antioxidant system	^89^
				immunosuppression	
				changes in the osmoregulation process	
diclofenac	laboratory	0.4 μg L^–1^	*A. altiparanae*	promotion of lipid peroxidation	^92^
				inhibition of antioxidant enzymes	
diclofenac	laboratory	3.08 mg L^–1^	*A. altiparanae*	SOD and GPx activity inhibition by DCF	^91^
				no effect on AChE activity	
ibuprofen diclofenac	laboratory	0.1, 1, 10, 100, and 1000 ng L^–1^	*A. lacustris*	reduction of *fsh*β and *lh*β gene expression	^93^
				decrease of 11-Ketotestosterone and T concentrations	
diclofenac	laboratory	0.2, 2.0, or 20 μg kg^–1^	*Hoplias malabaricus*	alteration of hematological parameters	^94,95^
				immunotoxic effects	
				oxidative stress	
diclofenac	laboratory	0.2, 2.0, and 20 μg L^–1^	*Rhamdia quelen*	nitric oxide production was reduced and alteration of 20 anti inflammatory-related proteins in plasma and kidney proteins	^101^

### 4.1 Endocrine Disruption on Brazilian Aquatic Species

The endocrine axes in vertebrates play a relevant role in metabolism and reproductive physiology.^102–104^ For example, the hypothalamic-pituitary-gonadal (HPG) axis is responsible for the control of reproduction, with gonadotropin-releasing hormone (GnRH) modulating the synthesis and release of follicle-stimulating hormone (FSH) and luteinizing hormone (LH) in the pituitary gland, which regulates the synthesis and release of sex steroid hormones by the gonads.^105^ The synthesized steroidal hormones 17β-Estradiol (E_2_) and testosterone (T) by the gonads ensure the positive or negative feedback loop in the HPG axis, which again controls the synthesis and release of gonadotropins and related steroidal hormones.^103,106^ HPG axis may experience interferences from the Hypothalamus-pituitary-thyroid (HPT) and Hypothalamus-pituitary-interrenal gland (HPI) axes in a crosstalk modulatory process.^104,107–109^ Such internal regulation supports the physiological adjustments of the animals to unfavorable environmental conditions during development, reproduction seasons, or stressful responses to predators or environmental pollution^109–111^ (Figure 2).

**Figure 2:**
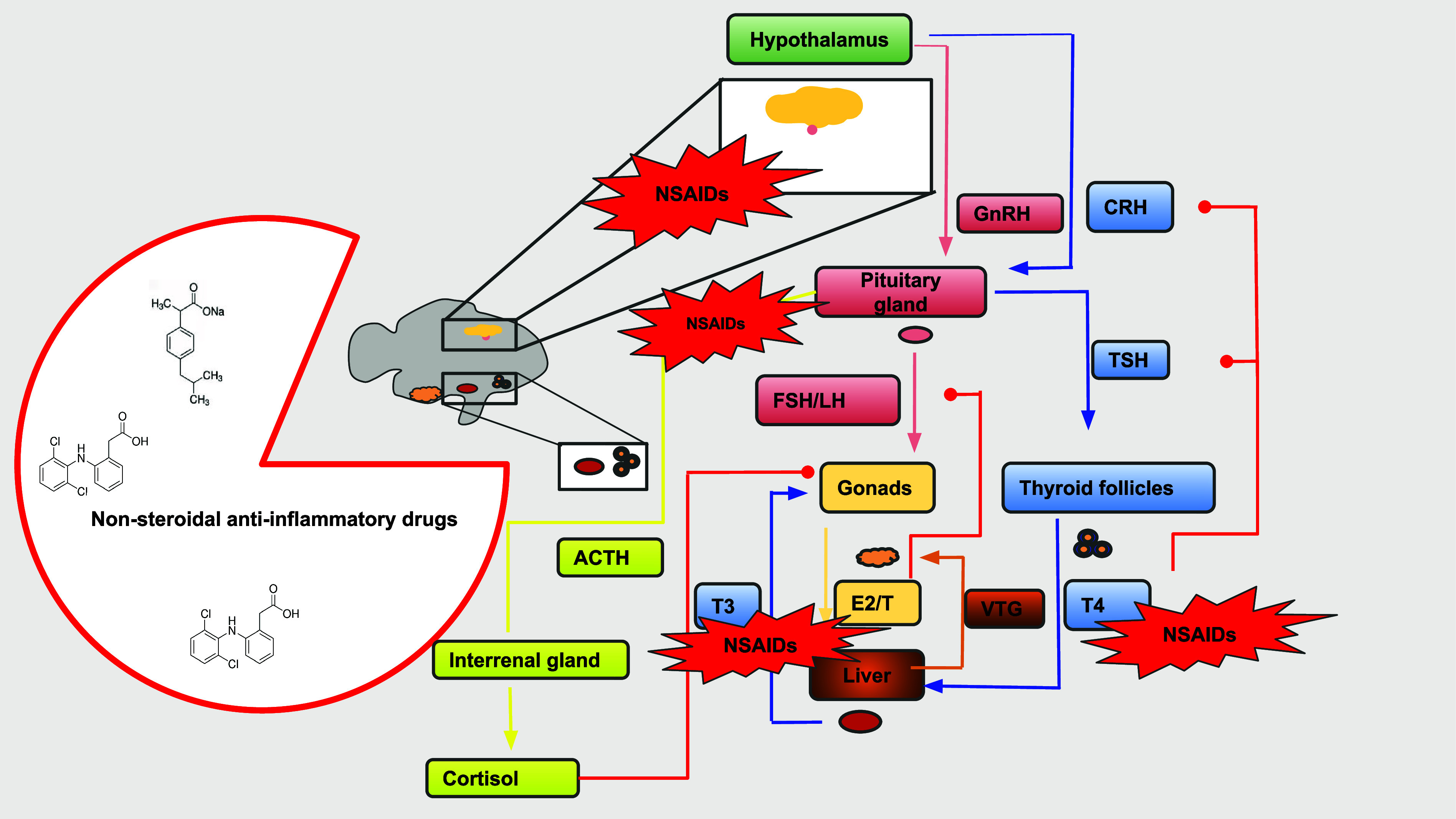
Main neuroendocrine axes on teleost fish, including hypothalamus-pituitary-gonads (HPG), hypothalamus-pituitary-thyroid (HPT), and hypothalamus-pituitary-interrenal gland (HPI) pathways, and possible pathways to endocrine disruption on Brazilian teleost fish.

During their lifespan, in natural ecosystems, many aquatic organisms are exposed to a wide range of chemicals with the potential capacity to induce endocrine disruption. These chemicals, including PhACs, represent a high potential to induce alterations in endocrine pathways.^102,112^ Much of those PhACs, including NSAIDs, represent a source of endocrine disruption to aquatic animals,^16^ and the effects extend to different endocrine axes, including HPG, HPT, and HPI axes. HPT and HPI axes are crucial players in the reproduction of aquatic species, and disrupting such systems could be dangerous to endemic species.^113^

The endocrine disruption effects caused by NSAID exposure are poorly evaluated on Brazilian aquatic species. Investigations regarding the major effects of NSAIDs are related to *A. lacustris* males in vivo studies^90–92^ and more recently in females of the same species,^86^ in vitro evaluations,^93^ and *R. quelen* in vivo assays.^88^ These investigations indicated that NSAIDs could induce changes in T and E_2_ circulating levels in *A. lacustris* males after high doses (3.06 mg L^–1^), but not in *A. lacustris* females exposed to a similar concentration range.^86^ Also, no alterations were observed at low doses (0.2, 2, and 20 μg L^–1^) as observed in *R. quelen* exposed for 21 days.^88^ Meanwhile, in vitro studies using testes explants from *A. lacustris* revealed that ibuprofen (0.1, 1, 10, 100, and 1000 ng L^–1^) decreased T concentration but did not alter E_2_ or diclofenac treatments.^93^ This study also observed a decrease in *lh*β and *fshl*β gene expression in pituitary explants. Thus, distinct effects are presented, but early inferences regarding NSAIDs’ endocrine-disruptive potential on native fish Brazilian species are limited since few fish species were investigated.

The literature elucidated that NSAIDs target not only COX enzymes, but modifications are present on aromatase enzyme activity,^114^ which is involved in hormone production and other biological processes. These results suggest a possible disturbance of E_2_ synthesis,^114^ which has implications for animal hormonal status as observed in the investigations using the native fish species, where steroid hormone concentration was reduced after NDSAIDs exposure,^90,93^ and also in *D. rerio* and *Oryzias latipes*.^114,115^ Several steroidal hormones play an important role in the reproduction, metabolism, and development of vertebrates, and a disruptive effect of chemicals such as PhACs could impact animal health and welfare.^112^ In fact, *D. rerio* exposed to 10, 100, or 1000 μg L^–1^ of ibuprofen for 14 days displayed a lower frequency of spawned eggs than nonexposed fish.^114^

Another challenge is that during NSAIDs exposures, many of the endocrine systems evaluated are the HPG axis,^88,90,93^ but recent investigations suggest different endocrine axes, such as the HPT axis, can be targeted by NSAIDs toxicity. Recently, *A. lacustris* females exposed acutely to diclofenac (3.06 mg L^–1^) and ibuprofen (13.7 mg L^–1^) showed an increase in thyroxine (T4) concentration and reduction of T3 in 96 h.^86^ Also, long-term exposures to naproxen 0.1–100 μg L^–1^ elucidated a decrease of thyroid hormones, T4 and triiodothyronine (T3), and downregulation of genes thyroid stimulating hormone beta subunit (*tshb*) and iodothyronine deiodinase type 2 (*dio2*) in *D. rerio*.^116^

As illustrated in Figure 2 and based on recent scientific investigations, in fish, NSAID compounds may impact key endocrine end points, including hormones and genes related to HPG and HPT pathways, during short and long-term exposure.^90,93,114–116^ Unfortunately, the underlying mechanisms of such toxicity are still scarce, and there is a lack of information related to NSAID toxicity using multiple endocrine end points. Considering endemic species, and to the best of our knowledge, only a study using the *A. lacustris* species investigated the diclofenac and ibuprofen toxicity using multiple endocrine biomarkers of HPG, HPT, and HPI axis, steroid hormones (E_2_, T, and Cortisol). This study reported that hormonal end points were not significantly different, but thyroid hormones were altered, where T4 increased 24 h after ibuprofen exposure, while a reduction of T3 96 h after diclofenac treatment was observed.^86^ Even though some of the studies reported adverse effects using the milligram concentration range instead of the NSAIDs nanogram and microgram range reported in the Brazilian rivers. Such data opens new gaps to NSAIDs’ endocrine disruptive effects using environmentally relevant concentrations in the Brazilian aquatic biota, considering that indirect effects could affect metabolism and stress end points, and lately impact ecological end points, including survival and reproduction.

### 4.2 Oxidative stress

The oxidative stress concept is associated with the redox imbalance between antioxidant defenses and reactive oxygen species (ROS) production, resulting in cellular damage.^117,118^ The defensive biochemical profile of the antioxidant system contains specific biotransformation enzymes of phase I (e.g., cytochrome P450 family), phase II enzymes and cofactors (e.g., Glutathione S-transferases, UDP-glucuronyl transferases, Reduced and oxidized glutathione), and each phase contributes to the metabolization, and excretion process of many xenobiotics detected in waters.^117,118^ The antioxidant enzymes are commonly used as biomarkers for the risk assessment process involving the effects of several xenobiotics in organisms’ physiology after acute and chronic exposures and continuous exposure in the natural aquatic environment.^118,119^ Although the antioxidant system displays a strong physiological barrier against internal physiological disturbances caused by chemicals and other natural stressors, the disruption of such a system could result in an unbalanced redox and, lately, influence the production of reactive oxygen species, oxidizing different biomolecules, including lipids, proteins, and even DNA.^120^

Oxidative stress research has been present in several investigations, such as scientific reports and experimental assays that display the potential of NSAIDs to cause detrimental effects on the oxidative balance of aquatic biota.^16^ Nonetheless, original data using Brazilian native aquatic species is still limited, even considering the amount of information on other species.^17^ To the best of our knowledge, only the species *A. lacustris* ^91,92^ and *R. quelen* ^87–89^ were evaluated on the potential oxidative stress effects of NSAID exposure in native Brazilian fish species.

The exposure of *R. quelen* to diclofenac at environmentally relevant concentrations of 0.2, 2, and 20 μg L^–1^ increases superoxide dismutase (SOD) activity in the kidney.^87^ Another study observed an increase in the antioxidant defenses, such as glutathione S-transferase (GST), a reduction of SOD and catalase activity (CAT) in the liver and testes in the same species.^88^ Similar responses were observed after ibuprofen exposure to 0.1, 1, and 10 μg L^–1^, where the authors observed disturbance in the antioxidant system of *R. quelen*.^89^ Studies using *A. lacustris* exposed to diclofenac at environmentally relevant concentrations of 0.4 μg L^–1^ reported the promotion of lipid peroxidation and inhibition of antioxidant enzymes.^92^ The above studies described adverse effects in the oxidative stress biomarkers of endemic teleost species using environmentally relevant concentrations of NSAIDs found in Brazilian waters.^88,89,91,92^ As previously described in the present review, the concentration of NSAIDs in the Brazilian surface waters ranges from 0.76 ng L^–1^ ^64^ to 759,060 ng L^–1^ ^53^ and, thus, the results using native species that occurs in those rivers may display a possibility of impact in the natural environment which is polluted by the presence of NSAIDs in higher concentrations.

### 4.3 Development and Survival

Effects of NSAID exposure on the development of Brazilian neotropical fish species are nonexistent, and scientific studies have not been described, even considering the great biodiversity of aquatic species in Brazil.^85^ The survival end point was only used for lethal concentration estimation using *A. lacustris* species after exposure to diclofenac, resulting in an LC_50_ of 30.8 mg L^–1^ ^90^ and ibuprofen, resulting in an LC_50_ of 137 mg L^–1^.^86^ Such data was similar to studies using common carp (*Cyprinus carpio*) juveniles, where LC_50_ values were 175.6 mg L^–1^ for ibuprofen and 70.98 mg L^–1^ for diclofenac.^121^

However, studies investigating aquatic organisms exposed to diclofenac reveal distinct life-stage-dependent toxicity. For example, research on *Oryzias latipes* (Japanese medaka) reported an LC_50_ of 10 mg L^–1^,^122^ highlighting significant survival impacts at this concentration. Similarly, in brown trout (*Salmo trutta* *f. fario*), embryos exhibited notably lower sensitivity to diclofenac compared to juveniles, underscoring variability in pharmaceutical toxicity across developmental stages. These findings emphasize the importance of considering life-stage differences when assessing the ecological risks of NSAIDs.^123^

In the literature, the NSAIDs’ toxicity was also evaluated in the development of zebrafish *D. rerio*. The studies reported that ibuprofen and diclofenac exposure at 5, 50, and 500 μg L^–1^ displayed effects in hatching and motion of embryos, and the authors pointed out a threat to embryo development after exposure^124^ and in the cardiovascular development after exposure to 0.04 and 25.0 μg L^–1^ concentration range of diclofenac and ibuprofen.^125^ Similar malformation impacts were also described in zebrafish embryos after naproxen exposure to 1, 10, and 100 μg L^–1^.^126^ The teratogenic effects already described in standardized laboratory species reinforce the need to assess the effects of NSAIDs on the development of nontarget species, including the neotropical aquatic animals from Brazilian biota.

Despite the teratogenic effects reported in aquatic species, higher mortality was not expected as compared to other classes of chemicals, such as pesticides, so it is common to address the fact that they have no acute toxicity. Pharmaceuticals such as NSAIDs are designed for a specific pharmacological role during disease treatments^28^ and to demonstrate molecular stability inside the biological systems,^127^ so it is common to address that they have no acute toxicity. However, studies have shown that the toxicity of NSAIDs depends on several parameters, including life-stage, exposed organism, chemical structure, and exposure period.^16^

## 5 NSAID Mixture Effects and Bioconcentration

Another challenge to pharmaceutical investigations in Brazilian water matrices is the occurrence of a great amount of PhAC mixtures^128^ and the bioconcentration process. Current reviews and investigations highlighted the necessity of evaluating the effects of pollutant mixtures in biological systems at environmental concentrations to understand the possible interactions and toxicity to biota over time, which includes NSAID mixtures as well.^32^ Laboratory investigations using the teleost *Tinca tinca* during early life stages aimed to evaluate the capacity of NSAID mixtures, including diclofenac and ibuprofen (2, 20, and 60 μg L^–1^), to induce malformations and affect development, but after exposure, the abnormalities were only observed at the highest concentration of 60 μg L^–1^.^129^

Few studies have been addressing the PhACs mixture effects using endemic Brazilian neotropical species,^85,90–92^ even considering the ubiquitous presence of several PhACs and the susceptibility of animals, especially fish and other invertebrates.^128^ The results using *A. lacustris* have shown a lack of synergistic effects between NSAIDs mixtures when evaluating distinct endocrine end points in the exposed animals.^86,90^ Different interaction mechanisms between the chemicals may explain the lack of higher toxicity compared to the isolated compounds. Antagonistic interactions were observed in *C. carpio* exposed to diclofenac and ibuprofen mixtures;^121^ additional studies reported antagonistic responses in NSAIDs mixtures as the main predominant interaction result.^130^ Such issues highlight an additional complexity layer of the effects in the mixture investigations, which are crucial for understanding environmental toxicology studies.^131^

Regarding the bioconcentration of NSAIDs, bivalves are more commonly used to address the bioconcentration problems of NSAID compounds in marine waters.^132^ Recent investigations report bioconcentration factor of 0.07 for naproxen in three-spined stickleback (*Gasterosteus aculeatus*) whole fish after exposure to different concentration 18 to 1232 μg L^–1^, but the authors pointed out that naproxen is an environmentally better alternative than diclofenac which display a higher BCF of 0.3 ^133,134^ In fact, NSAIDs accumulation may display several toxic effects on a diversity of species, which includes vertebrates as teleosts and other invertebrates as arthropods and bivalves.^16,17,135^ In the downstream of a WWTP, NSAIDs such as diclofenac, ibuprofen, and naproxen were quantified in the bile of wild fish at maximum concentrations of 95, 34, and 32 ng mL^–1^, respectively.^136^ Thus, comparing the field and laboratory investigation, diclofenac displays a higher bioaccumulation process compared to other NSAIDs, which could result in higher toxicity compared to other NSAIDs.^132,133^

## 6 Ecotoxicological Risk Assessment of NSAIDs to Brazilian Species

The occurrence of NSAIDs across Brazilian surface waters and the effects observed in native species are not followed by an ecotoxicological risk assessment (ERA) for NSAIDs in the Brazilian territory. Therefore, a risk assessment framework was performed using ECOSAR short-term toxicity data (i.e., LC_50_ and E_50_) divided by the assessment factor (AF) of 1000 (short-term data) to determine the PNEC (predicted no-effect concentration) values for the different trophic levels (fish, daphnids, and algae).^137^ To estimate the PNEC values for the native species, the toxicity assessment containing the LC_50_ for the endemic species, *A. lacustris* and *R. quelen*, was obtained from previous studies.^86,90,138^ The risk quotient (RQ) was estimated by the measured environmental concentration (MEC) and the specific PNEC value of the NSAIDs for each organism (Table S1). Since the PNEC data for some NSAIDs are lacking, the missing values were described as NA in the table. Moreover, the interpretation of the RQ values was achieved following the rating risk where RQ ≥ 1 is considered high risk; 0.1 ≤ RQ < 1 for medium risk, and 0.01 < RQ < 0.1, low risk.^136,139^

Considering the contaminated water data set, ECOSAR and *A. lacustris* and *R. quelen* PNEC, the RQ values presented a low risk (Table S1). Still, this result may display limited information for the ERA evaluations due to restricted acute data for *A. lacustris* and *R. quelen* PNEC, and, thus, requesting future studies for a lower AF in order to reduce unambiguous extrapolations associated with laboratory experiments when compared to field studies. Nevertheless, the RQ values display a medium risk (RQ = 0.304) of diclofenac in the Surface water from the Tibagi River (PR) for *A. lacustris*, similar rating was also observed for the Surface water from the Stream of Onça (Três Lagoas-MS), showing a medium risk (RQ = 0.268) for *A. lacustris*. In the Northeast region, a high risk (RQ = 6.266) was observed for *A. lacustris* in the Surface water from Beberibe River Basin (Recife-PE). Finally, the highest risk coefficient for endemic species was observed in the Surface water from the São Francisco River (PE), and the RQ for diclofenac and ibuprofen was 24.6 and 5.7, respectively (Figure 3).

**Figure 3:**
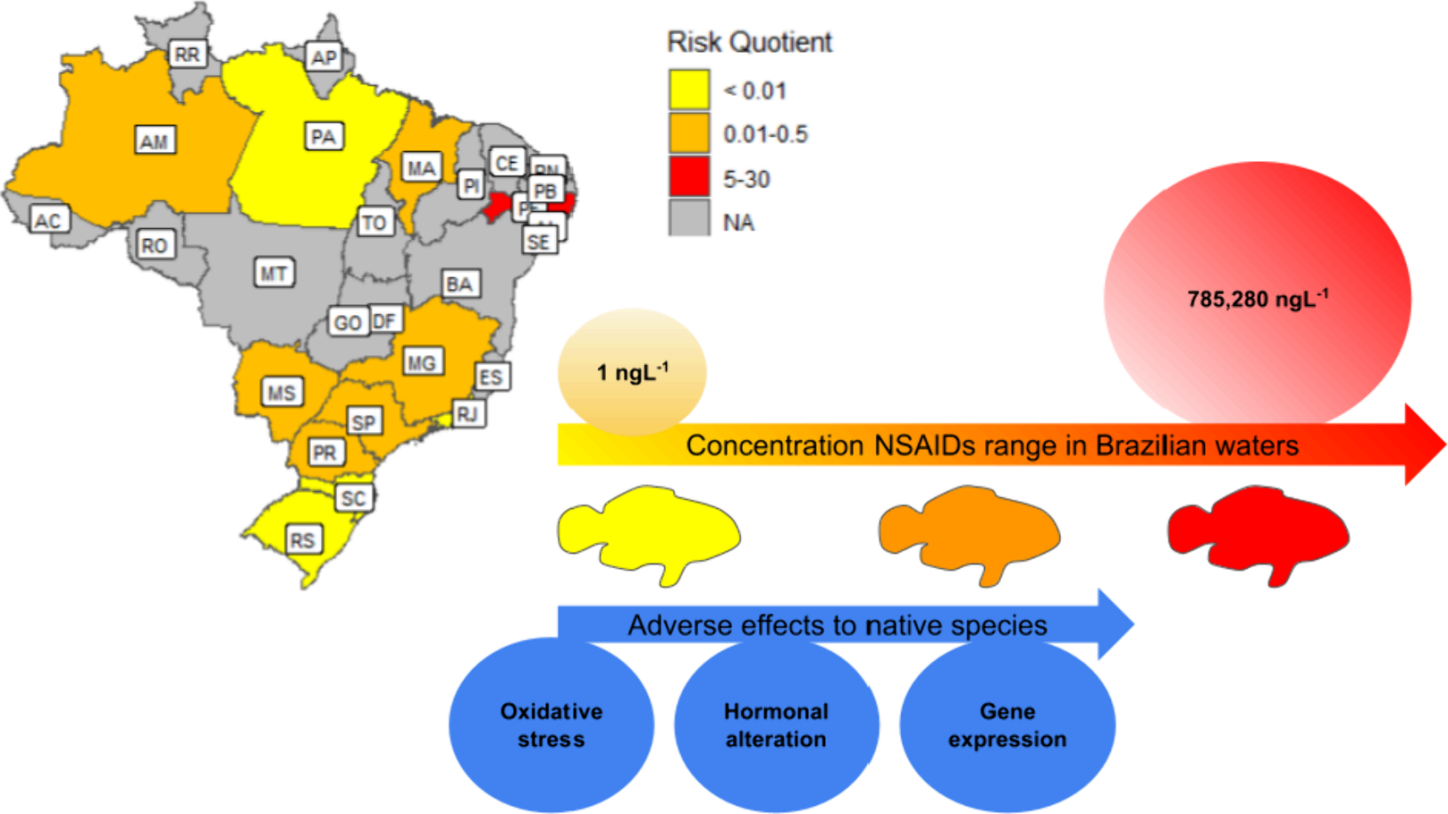
Risk Quotient (RQ) range in Brazilian territory associated with the NSAID concentration (1 to 785,280 ng L^–1^). The adverse effects were observed at lower concentrations. NA indicates the lack of data in the present geographic regions.

Diclofenac and ibuprofen showed the highest RQs for the species evaluated (Figure 4), reaching 29.4 and 157.1, respectively. Naproxen presented a medium risk for the majority of the studies, with the highest RQ observed for daphnids (RQ = 0.22). On the other hand, ketoprofen presented no risk for any of the three species, with a PNEC value, with RQ reaching 0.006.

**Figure 4:**
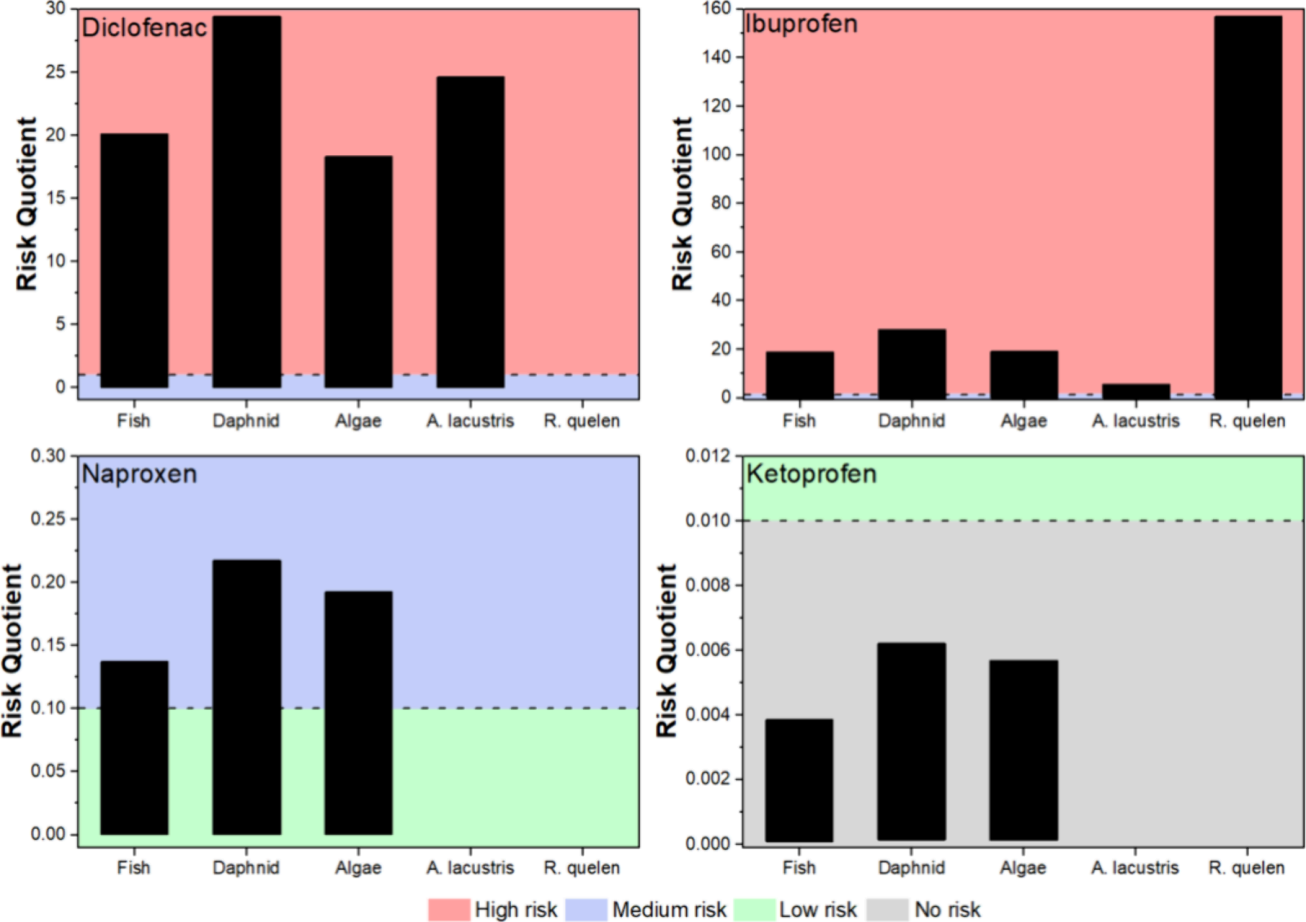
Risk Quotient for the NSAIDs considering the range of maximum concentrations found in the Brazilian studies.

Considering the broad NSAIDs concentration range starting in 1 to 785,280 ng L^–1^, much of the ERA using a limited number of species may not give clear insights about the RQ to nontarget species in the Brazilian territory. The initial laboratory experiments using endemic species, such as *A. lacustris* and *R. quelen*, suggest adverse effects in the redox balance and physiological end points, including metabolism and reproduction, even at low concentrations and short exposure periods.^88,91,92^ Despite the logistical challenges and scarce biological data related to endemic species, chronic experiments may seem more suitable for understanding the risks of NSAIDs to the aquatic environment.

The increasing amount of data in the literature indicates several impacts of NSAIDs exposure to juveniles and different animals in the aquatic biota.^16^ Moreover, many of the metabolites of NSAIDs are also detected in the natural environment, and investigations suggest that they can be more toxic compared to the parental compounds,^37^ but the ecotoxicological studies are still missing, comparing these additional effects. Even in invertebrate animals, the toxic mechanisms of NSAIDs chemicals in different species are still lacking when considering the great importance of invertebrates to aquatic environments.^17^ Thus, to extrapolate the effects of NSAIDs, relevant species of each trophic level must be applied to understand their toxicity, including organisms with ecological relevance.

## 7 Conclusions, Remarks, and Future Directions

The present review aimed to provide the main relevant information regarding the occurrence of NSAIDs in the aquatic environment of Brazil and the biological effects caused by NSAIDs after long- and short-term exposures. NSAIDs are one of the most prescribed pharmaceuticals, and their ubiquitous presence is a topic of emerging concern, mainly in countries such as Brazil, where WWTPs are not able to remove much of the PhACs detected in the waste effluents. The majority of the biological information on the NSAIDs’ impacts is limited to 3 neotropical teleost species, which could be linked to many factors, such as impaired logistics to assess laboratory services, insufficient scientific financial funds, and a lack of basic biology knowledge of many aquatic species still unknown to the scientific community. Therefore, it is essential to develop new methodologies to evaluate and monitor the effects of NSAIDs on other aquatic species.

Regarding concerns, the limited information in the literature about the effects of NSAIDs is far from showing the total ecotoxicological impacts, and considering the absence of legislation, the limits and possible effects could be addressed in new investigations. Brazilian ecosystems account for great global biodiversity, and the presence of PhACs such as NSAIDs brings a new challenge to investigations into the long-term effects on environmental balance and human health. The focus on new species and key models to assess these adverse effects should be prioritized. Till now, approaches including hormone measurements and redox balance evaluations have been used, but far other mechanisms of toxicity after NSAID exposure, risk assessment tools, such as ecological end points, could be good estimators of the hazard impacts of NSAIDs.

Another approach to NSAID investigations is the use of different physiological end points to assess NSAID toxicity, using long-term exposures to evaluate HPT axis responses, generational studies, metabolic disorders, and morphological alterations. The environmental health impacts on biodiversity and environmental quality are still unknown due to the absence of reliable data in several regions of Brazil’s territory, scarce information on the river’s contamination, and monitoring programs are almost nonexistent. This should be considered a challenge and a call for environmental and health investigations. The effects already described in the literature using native species are an alert to regulatory authorities and the general population about the risk of NSAIDs to environmental health and the necessity to establish guiding levels of risk in waters.

Despite significant research efforts aimed at understanding the environmental occurrence of NSAIDs and their negative impacts on nontarget species, the translation of academic findings into robust environmental legislation remains slow and bureaucratic, particularly in developing countries across Latin America. Given the widespread consumption of NSAIDs, their documented presence in Brazilian and Latin American waters, and the demonstrated biological effects on nontarget aquatic species, it is essential to recognize the vital role of society in environmental protection and in safeguarding both human and animal welfare. Therefore, the adoption of a comprehensive One Health approach to address NSAID contamination is urgently needed.

## Supplementary Material



## Notes

Ecotoxicological risk assessment of NSAIDs to Brazilian species (PDF)
