# Supplementary material for: Review of the Nonsteroidal Anti-Inflammatory Drug Consumption, Occurrence, Potential Impacts on Environmental Health, and Insights into Regulatory Decision-Making Brazilian Aquatic Ecosystems
Source: ACS Omega. 2025 Jun 13;10(25):26250–65. doi: 10.1021/acsomega.5c01916 (PMC12224100; doi:10.1021/acsomega.5c01916)
Supplement: Supplementary file 1 [file ao5c01916_si_001.pdf]

Supplementary Material

Review of the Nonsteroidal Anti-Inflammatory Drug Consumption, Occurrence, Potential Impacts on Environmental Health, and Insights into Regulatory Decision-Making Brazilian Aquatic Ecosystems

Filipe G.A. Godoi[a], Mariana A. Dias[b], Cassiana C. Montagner[b], Fabiana L. Lo Nostro[c], Renata G. Moreira[a]

[a]Departamento de Fisiologia, Instituto de Biociências, Universidade de São Paulo, Rua do Matão, Trav.14, n° 321, 05508-090 São Paulo, SP, Brazil
[b]Laboratório de Química Ambiental, Departamento de Química Analítica, Instituto de Química - UNICAMP, Rua Monteiro Lobado 270, 13083-862, Campinas SP, Brazil
[c]Lab. de Ecotoxicología Acuática, Departamento de Biodiversidad y Biología Experimental, Facultad de Ciencias Exactas y Naturales, Universidad de Buenos Aires & IBBEA, CONICET-UBA, Ciudad Universitaria C1428EHA, 1429, Buenos Aires, Argentina

* Corresponding author: Filipe G.A. Godoi, godoifga@usp.br

Table S1. Ecotoxicological risk assessment of nonsteroidal anti-inflammatory (NSAID) in Brazilian surface waters.

Sampling site	Chemical	Max Concentration (ng L-1)	Fish ECOSAR PNEC (ng L-1)	Daphnid ECOSAR PNEC (ng L-1)	Algae ECOSAR PNEC (ng L-1)	*Astyanax lacustris* PNEC (ng L-1)	*Rhamdia quelen* PNEC (ng L-1)	RQ fish	RQ Daphnid	RQ algae	RQ *Astyanax lacustris*	RQ *Rhandia quelen*
Surface water from Piraí and Jundiaí rivers (Jundiaí River Basin-SP)	Diclofenac	328.5	37700	25800	41400	30800	NA	0.009	0.013	0.008	0.011	NA
	Ibuprofen	208.2	41600	27800	41100	137000	5000	0.005	0.007	0.005	0.002	0.042
	Naproxen	98.6	193000	122000	138000	NA	NA	0.001	0.001	0.001	NA	NA
Surface water from Piraí and Jundiaí rivers (Jundiaí River Basin-SP)	Diclofenac	364	37700	25800	41400	30800	NA	0.010	0.014	0.009	0.012	NA
	Ibuprofen	373	41600	27800	41100	137000	5000	0.009	0.013	0.009	0.003	0.075
	Naproxen	145	193000	122000	138000	NA	NA	0.001	0.001	0.001	NA	NA

Sampling site	Chemical	Max Concentration (ng L-1)	Fish ECOSAR PNEC (ng L-1)	Daphnid ECOSAR PNEC (ng L-1)	Algae ECOSAR PNEC (ng L-1)	*Astyanax lacustris* PNEC (ng L-1)	*Rhamdia quelen* PNEC (ng L-1)	RQ fish	RQ Daphnid	RQ algae	RQ *Astyanax lacustris*	RQ *Rhandia quelen*
Surface water from urban drainage channel in São Vicente Island (SP)	Diclofenac	2.5	37700	25800	41400	30800	NA	0.000	0.000	0.000	0.000	NA
Surface water from urban drainage channel in Santos beaches (Santos-SP)	Diclofenac	3.5	37700	25800	41400	30800	NA	0.000	0.000	0.000	0.000	NA
Surface water from urban drainage channel in Guarujá beach (SP)	Diclofenac	79.8	37700	25800	41400	30800	NA	0.002	0.003	0.002	0.003	NA
Surface- and bottom water around the coastal submarine sewage outfall in Guarujá (Santos-SP)	Diclofenac	85.7	37700	25800	41400	30800	NA	0.002	0.003	0.002	0.003	NA
Surface water from coastal rivers in São Paulo coast (SP)	Diclofenac	3.93	37700	25800	41400	30800	NA	0.000	0.000	0.000	0.000	NA
Surface- and bottom water from Santos Bay (Santos-SP)	Diclofenac	19.4	37700	25800	41400	30800	NA	0.001	0.001	0.000	0.001	NA
Surface- and bottom water from Santos Bay (Santos-SP)	Ibuprofen	2094.4	41600	27800	41100	137000	5000	0.050	0.075	0.051	0.015	0.419

Sampling site	Chemical	Max Concentration (ng L-1)	Fish ECOSAR PNEC (ng L-1)	Daphnid ECOSAR PNEC (ng L-1)	Algae ECOSAR PNEC (ng L-1)	*Astyanax lacustris* PNEC (ng L-1)	*Rhamdia quelen* PNEC (ng L-1)	RQ fish	RQ Daphnid	RQ algae	RQ *Astyanax lacustris*	RQ *Rhandia quelen*
Surface water from Lobo reservoir (Itirapina-SP)	Diclofenac	50	37700	25800	41400	30800	NA	0.001	0.002	0.001	0.002	NA
	Ibuprofen	130	41600	27800	41100	137000	5000	0.003	0.005	0.003	0.001	0.026
	Naproxen	100	193000	122000	138000	NA	NA	0.001	0.001	0.001	NA	NA
Surface water from Monjolinho River (São Carlos-SP)	Diclofenac	385.6	37700	25800	41400	30800	NA	0.010	0.015	0.009	0.013	NA
	Ibuprofen	743.9	41600	27800	41100	137000	5000	0.018	0.027	0.018	0.005	0.149
	Naproxen	655.2	193000	122000	138000	NA	NA	0.003	0.005	0.005	NA	NA
Surface water from Itaipu-Piratininga coastal lagoons (Niterói-RJ)	Ibuprofen	37.6	41600	27800	41100	137000	5000	0.001	0.001	0.001	0.000	0.008
	Naproxen	22.5	193000	122000	138000	NA	NA	0.000	0.000	0.000	NA	NA
Surface water from rivers (Rio de Janeiro-RJ)	Diclofenac	220	37700	25800	41400	30800	NA	0.006	0.009	0.005	0.007	NA
Surface water from João Mendes River basin (Niterói-RJ)	Ibuprofen	10700	41600	27800	41100	137000	5000	0.257	0.385	0.260	0.078	2.140
Surface water from Paraopeba River Basin (MG)	Diclofenac	2625.7	37700	25800	41400	30800	NA	0.070	0.102	0.063	0.085	NA
Surface water from Paraopeba River Basin (MG)	Diclofenac	561	37700	25800	41400	30800	NA	0.015	0.022	0.014	0.018	NA
	Ibuprofen	1683.9	41600	27800	41100	137000	5000	0.040	0.061	0.041	0.012	0.337
	Naproxen	938.4	193000	122000	138000	NA	NA	0.005	0.008	0.007	NA	NA

Sampling site	Chemical	Max Concentration (ng L-1)	Fish ECOSAR PNEC (ng L-1)	Daphnid ECOSAR PNEC (ng L-1)	Algae ECOSAR PNEC (ng L-1)	*Astyanax lacustris* PNEC (ng L-1)	*Rhamdia quelen* PNEC (ng L-1)	RQ fish	RQ Daphnid	RQ algae	RQ *Astyanax lacustris*	RQ *Rhandia quelen*
Water from Drinking Water Treatment Plants (MG)	Ibuprofen	333	41600	27800	41100	137000	5000	0.008	0.012	0.008	0.002	0.067
	Ketoprofen	1020	264000	164000	179000	NA	NA	0.004	0.006	0.006	NA	NA
Surface water from water supply systems (Belo Horizonte-MG)	Diclofenac	1115.2	37700	25800	41400	30800	NA	0.030	0.043	0.027	0.036	NA
	Ibuprofen	1629.2	41600	27800	41100	137000	5000	0.039	0.059	0.040	0.012	0.326
	Naproxen	26566.2	193000	122000	138000	NA	NA	0.138	0.218	0.193	NA	NA
Surface water from Alto Iguaçu watershed (Curitiba-PR)	Diclofenac	285	37700	25800	41400	30800	NA	0.008	0.011	0.007	0.009	NA
	Ibuprofen	370	41600	27800	41100	137000	5000	0.009	0.013	0.009	0.003	0.074
Surface water from Iguaçu River (Curitiba-PR)	Ketoprofen	620	264000	164000	179000	NA	NA	0.002	0.004	0.003	NA	NA
	Naproxen	340	193000	122000	138000	NA	NA	0.002	0.003	0.002	NA	NA
Surface water from Tibagi River (PR)	Diclofenac	9375	37700	25800	41400	30800	NA	0.249	0.363	0.226	0.304	NA
	Naproxen	1566.4	193000	122000	138000	NA	NA	0.008	0.013	0.011	NA	NA
Surface water from Lake Guaíba (Porto Alegre-RS)	Diclofenac	107	37700	25800	41400	30800	NA	0.003	0.004	0.003	0.003	NA
	Ibuprofen	387	41600	27800	41100	137000	5000	0.009	0.014	0.009	0.003	0.077
	Ketoprofen	21	264000	164000	179000	NA	NA	0.000	0.000	0.000	NA	NA
	Naproxen	21	193000	122000	138000	NA	NA	0,000	0,000	0,000	NA	NA

Sampling site	Chemical	Max Concentration (ng L-1)	Fish ECOSAR PNEC (ng L-1)	Daphnid ECOSAR PNEC (ng L-1)	Algae ECOSAR PNEC (ng L-1)	*Astyanax lacustris* PNEC (ng L-1)	*Rhamdia quelen* PNEC (ng L-1)	RQ fish	RQ Daphnid	RQ algae	RQ *Astyanax lacustris*	RQ *Rhandia quelen*
Surface water from urban rivers (Porto Alegre-RS)	Diclofenac	1	37700	25800	41400	30800	NA	0.000	0.000	0.000	0.000	NA
	Naproxen	1	193000	122000	138000	NA	NA	0.000	0.000	0.000	NA	NA
Surface water from Cancela-Tamandaí and João Goulart watershed (Santa Maria-RS)	Ibuprofen	2710	41600	27800	41100	137000	5000	0.065	0.097	0.066	0.020	0.542
Estuarine water from Santa Catarina coastal area (SC)	Diclofenac	7.92	37700	25800	41400	30800	NA	0.000	0.000	0.000	0.000	NA
Surface water from Stream of Onça (Três Lagoas-MS)	Diclofenac	8250	37700	25800	41400	30800	NA	0.219	0.320	0.199	0.268	NA
	Naproxen	21285	193000	122000	138000	NA	NA	0.110	0.174	0.154	NA	NA
Surface water from streams in Dourados (MS)	Diclofenac	849	37700	25800	41400	30800	NA	0.023	0.033	0.021	0.028	
	Naproxen	681	193000	122000	138000	NA	NA	0.004	0.006	0.005	NA	NA
Drinking water (Brasília-DF)	Diclofenac	6.03	37700	25800	41400	30800	NA	0.000	0.000	0.000	0.000	NA
	Ibuprofen	4.8	41600	27800	41100	137000	5000	0.000	0.000	0.000	0.000	0.001
Surface water from Beberibe River Basin (Recife-PE)	Diclofenac	193000	37700	25800	41400	30800	NA	5.119	7.481	4.662	6.266	NA
Surface water from São Francisco River (PE)	Diclofenac	759060	37700	25800	41400	30800	NA	20.134	29.421	18.335	24.645	
	Ibuprofen	785280	41600	27800	41100	137000	5000	18.877	28.247	19.107	5.732	157.056

Sampling site	Chemical	Max Concentration (ng L-1)	Fish ECOSAR PNEC (ng L-1)	Daphnid ECOSAR PNEC (ng L-1)	Algae ECOSAR PNEC (ng L-1)	*Astyanax lacustris* PNEC (ng L-1)	*Rhamdia quelen* PNEC (ng L-1)	RQ fish	RQ Daphnid	RQ algae	RQ *Astyanax lacustris*	RQ *Rhandia quelen*
Surface water from Anil and Bacanga rivers (São Luis-MA)	Diclofenac	463	37700	25800	41400	30800	NA	0.012	0.018	0.011	0.015	
	Ibuprofen	320	41600	27800	41100	137000	5000	0.008	0.012	0.008	0.002	0.064
Surface water from Igarapé do 40, Igarapé Mindu and Rio Negro (Manaus-AM)	Diclofenac	785	37700	25800	41400	30800	NA	0.021	0.030	0.019	0.025	NA
Water from Bolonha Water Treatment Plant (Belém-PA)	Ibuprofen	9.1	41600	27800	41100	137000	5000	0.000	0.000	0.000	0.000	0.002
	Naproxen	351.8	193000	122000	138000	NA	NA	0.002	0.003	0.003	NA	NA
